# Mental health and related influencing factors among rural elderly in 14 poverty state counties of Chongqing, Southwest China: a cross-sectional study

**DOI:** 10.1186/s12199-020-00887-0

**Published:** 2020-09-10

**Authors:** Yin Yang, Hui Deng, Qingqing Yang, Xianbin Ding, Deqiang Mao, Xiaosong Ma, Bangzhong Xiao, Zhaohui Zhong

**Affiliations:** 1 Department of Epidemiology, School of Public Health and Management, Research Center for Medicine and Social Development, Innovation Center for Social Risk Governance in Health, Chongqing Medical University, YiXueYuan Road, YuZhong District, Chongqing, 400016 China; 2 The First Affiliated Hospital of Chongqing Medical University, Chongqing, China; 3 Chongqing Preventive Medicine Association, Chongqing, China; 4 Chongqing Municipal Center for Disease Control and Prevention, Chongqing, China

**Keywords:** Mental health, Poverty state counties, Chinese elderly, K10

## Abstract

**Background:**

China has the largest elderly population in the world; little attention has been paid to the mental health of elderly in areas of extreme poverty. This is the first study to investigate the mental health of the rural elderly in poverty state counties in Chongqing and was part of the Chongqing 2018 health literacy promotion project.

**Methods:**

In 2019, a cross-sectional study was conducted to investigate the mental health status of the rural elderly in fourteen poverty state counties of Chongqing, in which a total of 1400 elderly aged ≥ 65 years were interviewed, where mental health status was measured by the ten-item Kessler10 (K10) scale. Ordered multivariate logistic regression was performed to evaluate the influencing factors related to mental health of the elderly in these areas.

**Results:**

The average score of K10 in 14 poverty state counties was 17.40 ± 6.31, 47.6% was labeled as good, 30.2% was moderate, 17.0% was poor, and lastly 5.1% was bad, and the mental health status of the elderly in the northeastern wing of Chongqing was better than the one in the southeastern wing of Chongqing. A worse self-rated health was the risk factor for mental health both in the northeastern and southeastern wings of Chongqing (all *P* < 0.001). Lower education level (OR (95% CI) = 1.45 (1.12–1.87), *P* = 0.004) was a risk factor in the northeastern wing, whereas older age (OR (95% CI) = 1.33 (1.13–1.56), *P* = 0.001) was a risk factors in the southeastern wing.

**Conclusions:**

The results showed that mental health of the elderly in poverty state counties was poor, especially in the southeastern wing of Chongqing. Particular attention needs to be paid to the males who were less educated, older, and single; female with lower annual per capital income; and especially the elderly with poor self-rated health.

## Introduction

Population aging is becoming an important public health problem in the world. Internationally, countries that typically have more than 10% of the population over the age of 60 are called an aging society. Due to the “family planning” policy over the past 30 years and also to the increase life expectancy, the proportion of Chinese elderly population has increased rapidly over the past few decades [1]. By 2017, there were 241 million old adults aged over 60 years in China, these accounting for 17.3% of the total population [2]. At the end of 2018, this number exceeded 249 million, accounting for 17.9% of the total population. Moreover, the population aged 65 and above reached 160 million, accounting for 11.9%. So, in fact, the aging of Chinese population is accelerating. According to the national office on aging, the number of elderly people aged 60 or above will reach 300 million by 2025. By 2033, it will be more than 400 million, and by 2050, it will reach 487 million, about a third of the total Chinese population [3].

As the most populous developing country, China has the largest elderly population in the world. Due to the increase of chronic diseases, decrease in physical function, cognitive impairment, loneliness, and widowhood, old people are more likely to have mental health problems [4–8]. Approximately 15% of adults aged 60 and over suffer from a mental disorder, the most common of which are depression and anxiety, and around a quarter of deaths from self-harm are among people aged 60 or above [9]. A previous study in China found that 27.8% of old people had psychological disorders [10], which aroused our attention.

Compared to the urban elderly, the mental health of rural elderly is more serious and neglected more easily [10]. First, there are deficiencies in rural infrastructure construction, medical treatment, and insurance system. Also, the use of primary health services by rural elderly is poor [11], and elderly in rural areas have a higher risk of chronic disease [12]. In addition, the mental health of rural residents has long been neglected by researchers and policy makers. Moreover, because of their relatively low economy and educational level, especially regarding mental health knowledge, it is difficult for them to discover their potential mental health risks like urban residents and take the initiative to seek medical or psychological assistance [13, 14].

Previous studies about mental health of the elderly in rural areas have been reported, most of them focused on the general rural elderly and the comparison of rural and urban difference [8, 10, 12]. However, little is known about the mental health of the rural elderly in poverty state counties of China. International studies have showed that the elderly with lower income had worse functional impairment and worse mental health than those who reported higher income [15, 16]. Also, a recent study in China showed that socioeconomic status was closely related to the mental health of elderly [17]. Compared with the general rural elderly, the elderly in rural areas of poverty state counties have lower cultural and economic conditions and also lower medical insurance. Although, in recent years, the government has paid more and more attention to the economic development of the poverty state counties, still the mental health of the elderly in these places is neglected. So far, no research had reported the mental health of elderly in these places. Therefore, in order to pay more attention to the mental health of the elderly in poverty state counties of China, we investigated the mental health status of the rural elderly in fourteen poverty state counties in Chongqing, southwest China, to provide relevant data for government decision makers.

## Methods

### Study location and research participants

#### Poverty state counties

**Fig. 1:**
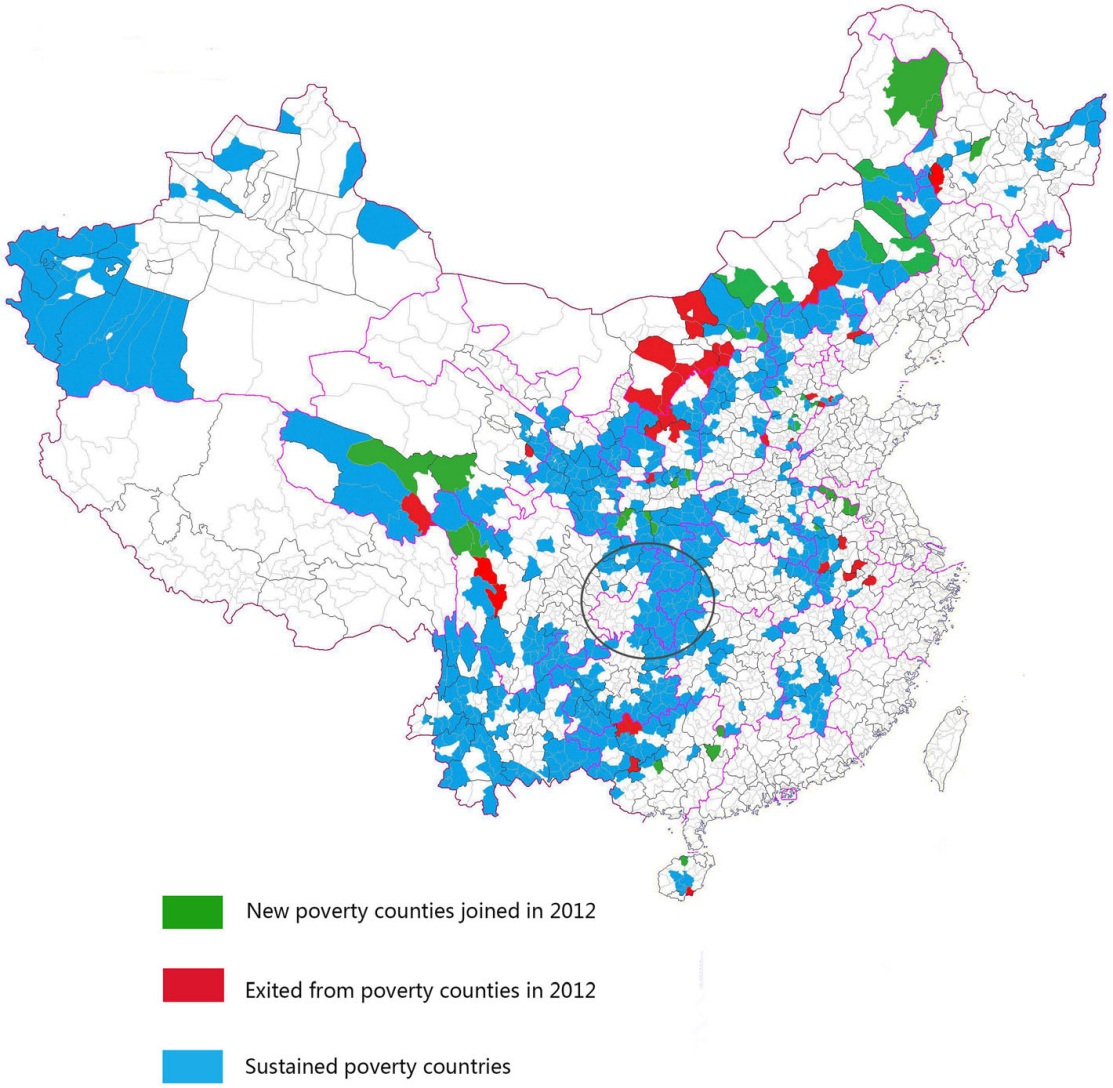
Location distribution of poverty state counties in China (the map is not fully displayed)

In 1985, the counties with a per capita annual income of less than 150 CNY were defined as poverty state counties. In 1992, among these counties, those with a per capita annual income of more than 700 CNY were excluded from poverty state counties, and other counties with a per capita annual income of less than 400 CNY were included in the poverty state counties. In 2012, poverty state counties were adjusted again; however, this time, no clear cut-offs were used in defining the counties (38 counties were discharged, and 38 new counties were added, so the total number remained unchanged) (Fig. 1). These places are located mainly in the central region, the mountainous regions in the west, and the southwestern and northeastern regions of China (Fig. 1). They are characterized by harsh natural environment, weak infrastructure, low basic social services, such as education and health care, low fiscal revenues, and seriously insufficient public basic investments.

Chongqing is one of the rapidly developing cities located in Southwest China with a population of approximately 31.02 million and area of 82,402 km^2^. Elderly aged over 60 years old account for 20.3% of the total population [18]. There are thirty-eight county-level administrative regions in Chongqing, of which fourteen are poverty state counties. This study was part of the Chongqing 2018 health literacy promotion action project.

**Fig. 2:**
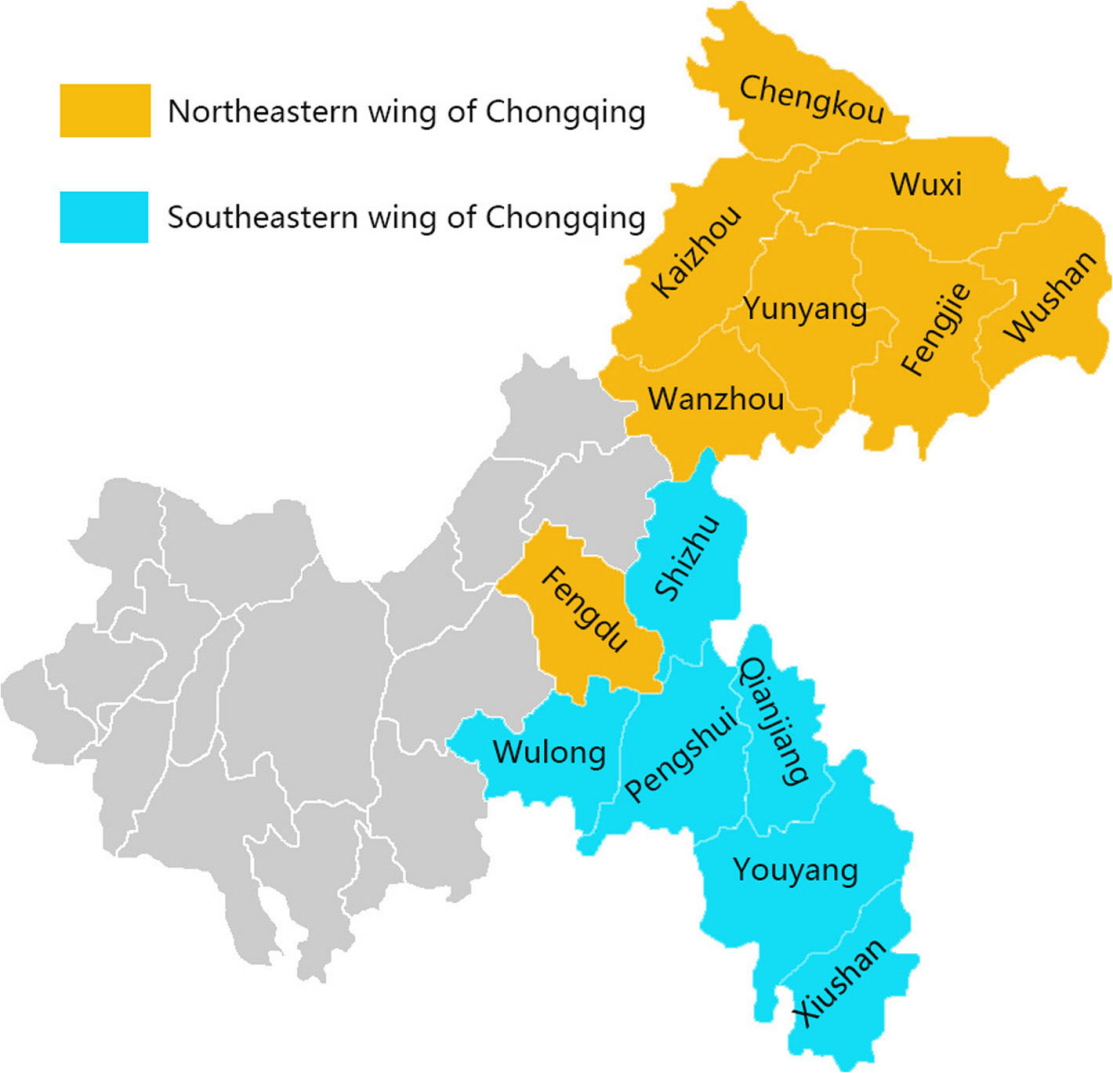
Location distribution of 14 poverty state counties in Chongqing

According to the characteristics of socioeconomic development and regional distribution, Chongqing is divided into three parts: urban economic circle, northeastern wing of Chongqing, and southeastern wing of Chongqing (Fig. 2). Between the 14 poverty state counties in Chongqing, 8 belong to the northeastern wing of Chongqing, from which the majority are of Han ethnicity, and 6 belong to the southeastern wing of Chongqing, where other ethnic minorities can be found. Considering the differences of ethnic cultures, living habits, and religious faiths between two rural areas, we compared the mental health of the two areas and discussed them separately.

Sampling consists of 2 stages. In the first stage, 18 towns of the 14 poverty state counties were chosen through purposive sampling as these towns are in deep poverty. In the second stage, 100 elderly of over 65 years old of rural areas were selected (convenient sampling) from each poverty state county, from the previously selected 18 towns of deep poverty (Supplementary figure 1). Elderly who had severe cognitive impairment, Alzheimer’s, or severely deaf were excluded. A total of 1400 elderly were interviewed, of those, 168 were excluded for not completing the questionnaire or for making obvious logical errors. This resulted in 1232 available questionnaires, with an effective rate of 88.0%. Among them, 685 were from the northeastern wing and 547 were from southeastern wing of Chongqing. This study was approved by Chongqing Health Bureau and Chongqing Medical University, and considering the educational level of the elderly (many old people cannot write), we only obtained the oral informed consent of the elderly.

### Data collection and measures

The investigators interviewed participants face-to-face to collect information between February and May 2019. All of the investigators were employees from the Centers for Disease Control and Prevention of their local counties, who also had a 1-day training at Chongqing Municipality Center for Disease Control and Prevention for this survey.

The mental health of rural elderly was measured by the Kessler Psychological Distress Scale (K10) [19]. This scale is a non-specific and concise screening method regarding psychological distress. It contains 10 items, where each has five response categories: never, rarely/seldom, sometimes, most of the time, and always. These were scored from one to five. The total minimum score is 10, indicating no distress, and the total maximum score being 50, indicating severe distress [20]. And in China, this scale was divided into 4 levels: 0 to 15 points (good mental health), 16 to 21 points (moderate mental health), 22 to 29 (poor mental health), 30 to 50 (bad mental health), this scale has good reliability and validity among the elderly in rural China [21].

We also collected some demographic information about age, gender, race, marital status, family income, degree of education, self-rated health, chronic diseases, and other information. Self-rated health was measured by a single item asking: “How do you evaluate your health at this time?” Responses were a five-point scale from “very bad” (1) to “very good” (5), where a higher score meant a better health. Chronic diseases were measured by asking: “Do you have chronic diseases diagnosed by doctors?” If the answer was yes, they were led to choose the type of chronic disease, such as anemia, diabetes, hyperlipidemia, cataract, COPD, arthritis, hypertension, ischemic heart disease, cerebrovascular disease, nasopharyngitis, chronic nephritis, and others.

### Quality control

All investigators were trained uniformly to have strict investigation standards. During the investigation, the quality control personnel of each county supervised the whole investigation process and checked the logic errors of the questionnaire. A real-time double-entry method was used for data entry verification.

### Statistical analysis

We used the Epidata3.1 software for data entry. The general characteristics of participants were presented as number and percentages. Chi-square test was performed to compare the demographic characteristic of the two regions. Ordinal logit regression analyses were used to compare the mental health status between northeastern wing and southeastern wing of Chongqing and the independent variables related to mental health. The results were reported as odds ratios (ORs) with 95% confidence intervals (CIs). Before the regression analysis, we checked the multicollinearity among independent variables, and no issues were found on multicollinearity among variables. Statistical analysis was performed using IBM SPSS version 22.0. A value of *P* < 0.05 was considered significant.

## Results

### Basic information of participants

The distribution of socio-demographic characteristics is summarized in Table 1. A total of 1400 elderly were interviewed. After confirming the validity of each questionnaire, only 1232 remained, of which 685 were from the northeastern wing and 547 were from southeastern wing of Chongqing. There were 669 males (54.3%) and 563 females (45.7%), and the majority of respondents were aged between 60 and 79 years (89.1%). Most were of Han ethnicity (69.4%), while 30.6% belonged to a minority. Overall, 90.4% of the study participants had an educational level of primary school or lower, with 35.5% illiterate. Married respondents comprised the largest percentage (75.6%). With regards to physical health status, 52.5% stated that they had no chronic disease, while 47.5% confirmed at least one chronic disease. About self-rated health, most respondents (44.2%) considered their current state of health to be moderate (Table 1). And the age, education level, income, and race were significant difference between the two wings (Table 1).

**Table 1:** Demographic characteristic of the elderly in poverty state counties of Chongqing

	Total *N* (%) (*N* = 1232)	Northeastern wing of Chongqing (*N* = 685)	Southeastern wing of Chongqing (*N* = 547)	*χ*^*2*^	*P*
**Sex**				1.898	0.168
Male	669 (54.3)	360	309		
Female	563 (45.7)	325	238		
**Age (year)**				11.860	0.008*
65–69	481 (39.0)	288	193		
70–74	398 (32.3)	226	172		
75–79	219 (17.8)	110	109		
>= 80	134 (10.9)	61	73		
**Race**				660.949	< 0.001**
Han population	855 (69.4)	682	173		
Minority	377 (30.6)	3	374		
**Education level**				8.805	0.032*
Illiteracy	437 (35.5)	263	174		
Primary school	676 (54.9)	356	320		
Junior middle school	106 (8.6)	56	50		
High school and above	13 (1.1)	10	3		
**Marital status**				0.209	0.976
Married	932 (75.6)	517	415		
Never married	25 (2.0)	14	11		
Divorced	12 (1.0)	6	6		
Widowed	263 (21.3)	148	115		
**Living arrangement**				0.539	0.463
Living alone	186 (15.1)	108	78		
Not live alone	1046 (84.9)	577	469		
**Annual per capital income**				47.841	< 0.001**
< 1000 CNY	162 (13.1)	70	92		
>= 1000 CNY	342 (27.8)	224	118		
>= 2000 CNY	261 (21.2)	138	123		
>= 3000 CNY	222 (18.0)	146	76		
>=5000 CNY	245 (19.9)	107	138		
**Chronic disease**				2.488	0.115
No	647 (52.5)	346	301		
Yes	585 (47.5)	339	246		
**Self-rated health**				7.821	0.098
Very good	97 (7.9)	66	31		
Fairly good	329 (26.7)	180	149		
Moderate	545 (44.2)	292	253		
Fairly bad	225 (18.3)	129	96		
Very bad	36 (2.9)	18	18		

*Chi-square test *P* < 0.05; **chi-square test *P* < 0.001

### Mental health of the elderly

What concerns mental health, the average score of K10 in the 14 poverty state counties was 17.40 ± 6.31. Four categories resulted from the K10 score: 47.6% was labeled as good, 30.2% was moderate, 17.0% was poor, and lastly, 5.1% was bad (Table 2). The mental health status of the elderly in northeastern wing was better than those in the southeastern wing of Chongqing before and after adjusting for confounders (before: OR 0.53, 95% CI 0.46–0.62; after: OR 0.61, 95% CI 0.48–0.78) (Table 3). Considering the differences of ethnic cultures, living habits, and religious faith between the two regions, we discussed them separately in the subsequent analysis.

**Table 2:** Mental health of the elderly in poverty state counties of Chongqing

	Category	Total *N* (%)	Northeastern wing of Chongqing *N* (%)	Southeastern wing of Chongqing *N* (%)
K10-score segments				
10–15	Good	587(47.6)	396 (57.8)	191 (34.9)
16–21	Moderate	372 (30.2)	167 (24.4)	205 (37.5)
22–29	Poor	210 (17.0)	93 (13.6)	117 (21.4)
30–50	Bad	63 (5.1)	29 (4.2)	34 (6.2)
Total	-	1232 (100.0)	685 (100.0)	547 (100.0)

**Table 3:** Comparison of mental health between Northeastern wing and Southeastern wing of Chongqing

	*B*	Wald	OR	95% CI	*P*
Northeastern wing^a^	− 0.630	64.352	0.53	0.46–0.62	**< 0.001**^**ζ**^
Northeastern wing^b^	− 0.647	62.046	0.52	0.45–0.61	**< 0.001**^**ζ**^
Northeastern wing^c^	− 0.496	17.382	0.61	0.48–0.78	**< 0.001**^**ζ**^

Dependent variable (K10-score segments): 1 = 0–15 points, 2 = 16–21 points, 3 = 22–29 points, 4 = 30–50 points. Southeastern wing of Chongqing was the reference group

^ζ^Ordinal logistic regression *P* < 0.001

^a^Univariate ordinal logistic regression

^b^Adjust for age, education level, and annual per capital income

^c^Adjust for age, education level, annual per capital income, and race

### Factors associated with mental health of elderly in poverty state counties of Chongqing

To explore the influence factors of mental health of elderly, socio-demographic and physical health variables were included into the regression analysis. The assignment of variables in the logistic regression analysis is shown in Table 4.

**Table 4:** Assignment of logistic regression analysis variables

Variables	Definition
**Dependent variable**	
Mental health	1 = good, 2 = moderately, 3 = poor, 4 = bad
**Independent variables**	
Sex	0 = male, 1 = female
Age (year)	1 = 65–69, 2 = 70–74, 3 = 75–79, 3 = ~ 80
Education level	1 = high school and above, 2 = junior middle school, 3 = primary school, 4 = illiteracy
Marital status	0 = single (never married, divorced, widowed), 1 = married
Living arrangement	0 = living alone, 1 = not living alone
Annual per capital income	1 = ~ 1000, 2 = ~ 2000, 3 = ~ 3000, 4 = ~ 4000, 5 = ~ 5000
Chronic disease	0 = yes, 1 = no
Race	0 = Han population, 1 = minority
Self-rated health	1 = very good, 2 = fairly good, 3 = moderately, 4 = fairly bad, 5 = very bad

The results showed that the worse self-rated health was the risk factor for psychological problems both in the northeastern wing and southeastern wing of Chongqing (all *P* < 0.001) (Table 5). In the northeastern wing of Chongqing, lower education level (OR 1.45, 95% CI 1.12–1.87) and single (OR 2.05, 95% CI 1.01–4.20) were risk factors for mental health of male, higher annual per capital income (OR 0.81, 95% CI 0.68–0.96) was a protect factor for mental health of female (Table 5), whereas in the southeastern wing an older age (OR 1.33, 95% CI 1.13–1.56) was a risk factor for mental health of male (Table 5).

**Table 5:** Ordered multivariate logistic regression of factors affecting the mental health of the elderly in poverty state counties of Chongqing

Variables	Total			Male			Female		
	OR	95% CI	*P*	OR	95% CI	*P*	OR	95% CI	*P*
**Northeastern wing of Chongqing**									
Age (year)	1.08	0.92–1.27	0.346	1.26	1.00–1.60	0.053	0.92	0.73–1.17	0.506
Education level	1.45	1.12–1.87	0.004*	1.54	1.10–2.17	0.012*	1.38	0.92–2.08	0.120
Annual per capital income	0.94	0.83–1.06	0.331	1.12	0.93–1.34	0.224	0.81	0.68–0.96	0.016*
Self-rated health	2.02	1.67–2.44	< 0.001**	2.45	1.84–3.26	< 0.001**	1.82	1.39–2.39	< 0.001**
Marital status									
Single	1.54	0.99–2.42	0.062	2.05	1.01–4.20	0.048*	1.37	0.76–2.48	0.291
Married	Reference			Reference			Reference		
Living arrangement									
Living alone	0.68	0.40–1.14	0.150	0.56	0.25–1.25	0.157	0.76	0.38–1.54	0.446
Not living alone	Reference			Reference			Reference		
Chronic disease									
Yes	1.04	0.74–1.45	0.837	0.92	0.56–1.52	0.750	1.06	0.67–1.68	0.808
No	Reference			Reference			Reference		
Sex									
Male	0.89	0.65–1.23	0.481	-	-	-	-	-	-
Female	Reference			-	-	-	-	-	-
**Southeastern wing of Chongqing**									
Age (years)	1.33	1.13–1.56	0.001*	1.33	1.07–1.65	0.009*	1.26	0.97–1.64	0.080
Education level	1.22	0.93–1.61	0.157	1.29	0.91–1.85	0.157	1.13	0.72–1.78	0.587
Annual per capital income	0.90	0.80–1.02	0.094	0.88	0.74–1.03	0.114	0.94	0.78–1.13	0.490
Self-rated health	1.58	1.26–1.98	< 0.001**	1.37	1.02–1.85	0.039*	1.97	1.39–2.80	< 0.001**
Marital status									
Single	0.80	0.49–1.31	0.374	0.71	0.35–1.41	0.329	0.97	0.48–1.98	0.936
Married	Reference			Reference			Reference		
Living arrangement									
Living alone	1.49	0.84–2.65	0.173	1.38	0.61–3.09	0.437	1.90	0.82–4.41	0.136
Not living alone	Reference			Reference			Reference		
Chronic disease									
Yes	0.91	0.63–1.32	0.633	1.21	0.74–1.98	0.444	0.58	0.32–1.03	0.061
No	Reference			Reference			Reference		
Race									
Han population	0.91	0.64–1.30	0.604	0.90	0.56–1.45	0.676	0.95	0.55–1.66	0.867
Minority	Reference			Reference			Reference		
Sex									
Male	0.81	0.58–1.14	0.231	-	-	-	-	-	-
Female	Reference			-	-	-	-	-	-

*Multivariate logistic regression *P* < 0.05; **Multivariate logistic regression *P* < 0.001

## Discussion

To our knowledge, this is the first study to investigate the mental health of the rural elderly in poverty state counties of Chongqing, aiming to understand the mental health status in these areas that were extremely poor and have been neglected, and providing relevant data for government’s health policy.

Our research found that from the total 14 poverty state counties, 47.6% accounted for a good mental health (K10 below 15), in northeastern wing 57.8%, while in southeastern wing of Chongqing just 34.9%. A study on elderly in another city, Weihai, China, showed that elderly with good mental health (K10 below 15) accounted for 70.8% [19]. Compared with foreign studies, the elderly in Australia who have a bad mental health status (K10 greater than 30) accounted for 1.7% [20], while elderly with bad mental health in Canada accounted for 3.6% [22]. However, in our study, this ratio accounted for 5.1% in 14 poverty state counties; in the northeastern wing of Chongqing, it accounted for 4.2%, while in the southeastern one it accounted for 6.2%. These data all indicate that the elderly in our study showed a mental health status that was even worse. Thus, it requires the government’s attention for devising the necessary measures. However, considering that the above references contained also urban populations, and that they conducted their study more in the past, so the comparison of the survey results only have a partial reference value.

We also found that education was correlated with mental health of elderly in the northeastern wing of Chongqing. Study participants with lower education were more likely to have higher risk of mental health problems. This finding reflects previous research on health literacy, where studies have found that highly educated people have more knowledge about strategies to stay healthy [23], which may be the reason for their better mental health status. However, in our study, most of the elderly have a low level of education, which is a high risk for their health.

In the southeastern wing of Chongqing, we found that mental health of the elderly worsened with the increase of age. As the age increased, the physical health of the elderly got worse, so they began to need more long-term services [17]. Social services and support in poverty state counties are usually not enough; also, the need of long-term services may bring them a greater economic burden. These may affect the mental health of the elderly. Moreover, older people are more likely to be widowed, and marriage is important for mental health, especially for the older generation [24]. Being without a spouse might lead to feelings of loneliness, which could increase the risk of depression.

Previous studies had reported that economic income is associated with mental health of the elderly [16, 25]. However, in our research, except the female of northeastern wing, income is not an influencing factor for the elderly in neither one of the regions. In rural areas of China, most of the elderly live with their families and they usually do not receive a fixed income, so it is likely that their children are bearing their cost of living. In addition, in recent years, with the financial aid of the government, the economy of the elderly has improved. This may be the reason why our research had different results.

Previous studies also showed that the number of chronic diseases can directly predict the health status of elderly people; it also indirectly affects perceived health through depression [26]. A community-based study of 1346 people in Taiwan showed that the number of physical illnesses was more important than physical functions (ability of daily life activities) in affecting the mental health of elderly [27]. Another study found that chronic diseases can significantly contribute to the development of mental health disorders over time, and also vice versa [5]. But at our study, we did not find a significant relationship between chronic diseases and mental health in neither one of the geographical areas. This may be due to our research subjects living in remote mountainous areas, where because of insufficient medical resources and lack of money most of the elderly with chronic diseases could not be diagnosed. So, just by self-reporting whether they have a chronic disease or not may bias the disease status. This could have reduced the association between chronic diseases and mental health in our study.

Similar to other findings [28, 29], we also found that elderly who had poor self-rated health were more likely to have worsened mental health status in both the northeastern and southeastern wings of Chongqing. Especially in northeastern wings of Chongqing, for every grade of self-reported health decline, the risk of poorer mental health in older people doubled. A meta-analysis also reported that a poor self-rated health status was more strongly associated with depression than the presence of chronic diseases [30]. A study in China including 16,074 subjects showed that residents with laboratory parameter abnormalities tended to have a poorer self-rated health; self-rated health is consistent with objective health status and can serve as a measure of health status in the general population [31]. Also, self-rated health is correlated with increased risk for mortality and life satisfaction of elderly [32, 33]. In our study, we considered that the self-rated health status may be a better indicator for residents’ objective health status than chronic diseases because if their access to medical care is limited, the state of the disease will always be underestimated. Therefore, we recommend that old adults with poor self-rated health should be screened for diseases and given more attention to their mental health.

This study has some limitations, first, due to the limitation of data acquisition, it is difficult to evaluate other factors that may have an impact on their mental health, such as their lifestyle habits (exercise, sleep) [34]. Second, we did not have a comprehensive understanding of the physical health status of the elderly. Some elderly may not know their disease because they did not seek medical treatment before, so the disease status may be underestimated. Third, our sample was from poverty state counties of Chongqing; therefore, generalization of our findings should be done with caution with regard to the poverty state counties of other cities of China. Moreover, our research had some selection bias: firstly, we excluded some hearing-impaired elderly who were unable to communicate with us. As the age increases, the hearing of the elderly will also gradually decline. So, the exclusion of hearing-impaired elderly may lead to the selection of younger and healthier elderly. However, the part that we chose to exclude was relatively small in number (mainly consisting of subjects with severe deafness), and also, by excluding these people, information bias was controlled. Secondly, because a large population flow towards urban areas. So, many elderly may be registered in the rural local area, but they have moved to urban areas so as to support their children. If random sampling was adopted, the rate of loss to follow-up would be too high, so the results of the study will not be able to accurately reflect the situation of the elderly living in this area. By using convenience sampling, we can ensure that the elderly in the selected survey sites are indeed properly surveyed. So, we adopted the method of convenient sampling, which may render our sample difficult for generalizing to the entire population. However, our sampling covered all the poverty state counties in Chongqing. In each county, we chose representative towns, where our researchers conducted door-to-door surveys in the town’s annexed villages. And the conditions of these annexed villages in same town were relatively homogeneous. The distribution of elderly people in these villages is random, so the samples were still representative to some extent.

Based on this survey, we have gained an overall understanding upon the mental health of the elderly in poverty state. We carried out corresponding health education publicity in these regions in the later period, to help them learn more about maintaining a proper mental and physical health. The government is currently training a group of experienced general practitioners (primary health care doctors) in these areas, including psychiatrists, and is constantly improving the personnel and equipment of township hospitals, to meet the health needs of people in these areas.

## Conclusions

Overall, the results showed that mental health of the elderly was poor, especially in the southeastern wing of Chongqing. Besides, factors affecting the mental health of the elderly are different in each of these regions. The government should not only pay attention to the economic development in poor areas, but also pay more attention to the mental health of the elderly in these areas. There is a high need of supplying medical resources to these places and also a high need to comprehensively consider various factors to prevent and treat the depression of the elderly. Particular attention needs to be paid to males who were less educated, older, and single; female with lower annual per capital income; and especially the elderly with poor self-rated health.

## Supplementary information

**Additional file 1: Supplementary Figure 1.** Flow chart for sampling subjects.

**Additional file 2.** Original data of 14 poverty state countries.

## Data Availability

All data generated or analyzed during this study are included in this published article and its supplementary information files.
