# Supplementary figures and images for: Mental health and related influencing factors among rural elderly in 14 poverty state counties of Chongqing, Southwest China: a cross-sectional study
Source: Environ Health Prev Med. 2020 Sep 10;25:51. doi: 10.1186/s12199-020-00887-0 (PMC7488569; doi:10.1186/s12199-020-00887-0)

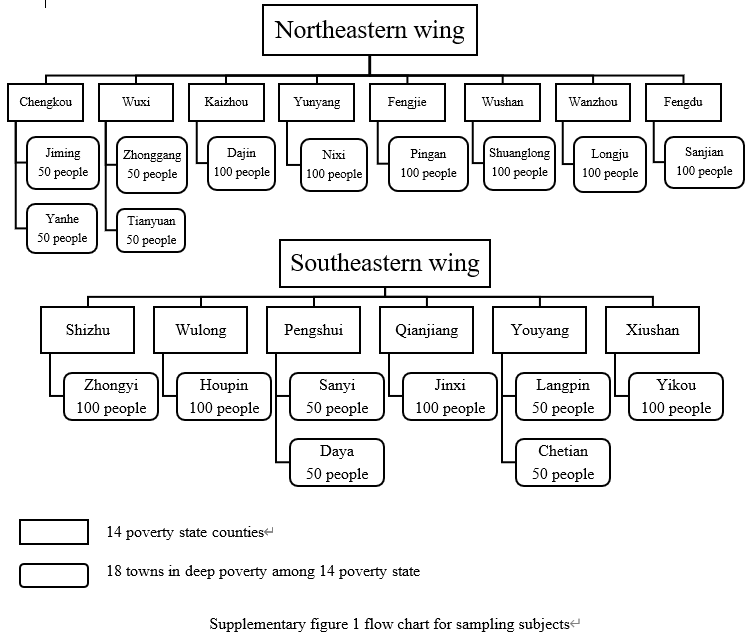

Supplement: Supplementary file 1 — Additional file 1: Supplementary Figure 1. Flow chart for sampling subjects. [file 12199_2020_887_MOESM1_ESM.png]
